# Thoracic skeletal muscle quantification using computed tomography and prognosis of elderly ICU patients

**DOI:** 10.1038/s41598-021-02853-4

**Published:** 2021-12-06

**Authors:** Sung Woo Moon, Song Yee Kim, Ji Soo Choi, Ah Young Leem, Su Hwan Lee, Moo Suk Park, Young Sam Kim, Kyung Soo Chung

**Affiliations:** 1 Division of Geriatrics and Integrated Medicine, Department of Internal Medicine, Yonsei University College of Medicine, Seodaemun-gu, Seoul, Republic of Korea; 2 Division of Pulmonary Medicine, Department of Internal Medicine, Yonsei University College of Medicine, 50 Yonsei-ro, Seodaemun-gu, Seoul, 120-752 Republic of Korea

**Keywords:** Geriatrics, Risk factors, Outcomes research

## Abstract

In elderly ICU patients, the prevalence of skeletal muscle loss is high. Longitudinal effect of thoracic muscles, especially in elderly ICU patients, are unclear although skeletal muscle loss is related with the short- and long-term outcomes. This study aimed to evaluate whether pectoralis muscle mass loss could be a predictor of prognosis in elderly ICU patients. We retrospectively evaluated 190 elderly (age > 70 years) patients admitted to the ICU. We measured the cross-sectional area (CSA) of the pectoralis muscle (PM_CSA_) at the fourth vertebral region. CT scans within two days before ICU admission were used for analysis. Mortality, prolonged mechanical ventilation, and longitudinal changes in Sequential Organ Failure Assessment (SOFA) scores were examined. PM_CSA_ below median was significantly related with prolonged ventilation (odds ratio 2.92) and a higher SOFA scores during the ICU stay (estimated mean = 0.94). PM_CSA_ below median was a significant risk for hospital mortality (hazards ratio 2.06). In elderly ICU patients, a low ICU admission PM_CSA_ was associated with prolonged ventilation, higher SOFA score during the ICU stay, and higher mortality. Adding thoracic skeletal muscle CSA at the time of ICU admission into consideration in deciding the therapeutic intensity in elderly ICU patients may help in making medical decisions.

## Introduction

As the population is rapidly aging, the proportion of elderly patients admitted to the intensive care unit (ICU) is increasing. Currently, the median age of critically ill patients approaches 65 years in many countries^1^. Elderly patients have a higher mortality rate in the ICU, and patients surviving the ICU often suffer from sequalae^2^. Caring for older patients frequently poses ethical and practical challenges, both prior to and during ICU admission^3^. In order to determine the level of treatment for elderly patients, we need to consider functional recovery as well as their chances of survival in the ICU.

Loss of muscle mass is a component of sarcopenia and is associated with a risk of adverse outcomes, such as disability, poor quality of life, and death^4^. Loss of muscle mass is also related to negative outcomes in patients with various lung diseases, cholangiocarcinoma, and breast cancer^5–8^. Bioelectrical impedance analysis, dual energy X-ray absorptiometry, magnetic resonance imaging, and B-mode ultrasound are widely used to quantify both total and local skeletal muscle mass^4^. Measurement of the cross-sectional area (CSA) of skeletal muscles on single-slice axial computed tomography (CT) is an alternative method to assess local skeletal muscle mass^9^. Correlations have been found between the muscle CSA at a single thoracic level on chest CT and total muscle volume^10–12^. Previous studies reported that at the level of the fourth vertebral region, the CSAs of the skeletal muscles are associated with frailty markers in lung transplantation and cardiac surgery patients^8,13^. Based on these studies, we have shown that loss of skeletal muscle mass measured by quantifying the thoracic muscle is related to a higher mortality in idiopathic pulmonary fibrosis patients^14^.

A recent study showed that in ICU patients, the thoracic muscle mass is related to mortality^15^. Evidence suggests that skeletal muscle mass and strength decline in a linear fashion, with up to 50% of mass being lost by the eighth decade of life^16^. The consequences of loss of muscle mass can be severe in older adults, as the associated strength and functional declines can contribute to a number of adverse health outcomes^17–19^. In a previous study, the prevalence of sarcopenia was 71% among the elderly ICU patients^20^.

We, therefore, sought to determine whether a low skeletal muscle mass adversely affects the outcome in elderly patients admitted to the ICU. In this study, we focused on pectoralis muscles at the fourth vertebral region in elderly (aged > 70 years) ICU patients and hypothesized that skeletal muscle mass, measured by analyzing the major and minor pectorals muscles’ CSAs from chest CT images, could be a predictor of prognosis in elderly medical ICU patients.

## Results

### Baseline characteristics

Baseline characteristics of the study subjects stratified by PM_CSA_ are provided in Table 1. The distribution of the CSAs of the pectoralis muscles is shown in Supplementary Fig. 1, and sequential organ failure assessment (SOFA) scores during the follow-up period are shown in Supplementary Fig. 2. The cutoff value corresponds to the median of the CSA of the pectoralis muscles (male = 24.5 cm^2^, female = 18.3 cm^2^). Of the 190 patients, 113 were male and 77 were female. The most common reason for ICU admission was respiratory failure (48.9%). Overall, 121 patients (63.7%) were intubated, 43 (22.6%) died in the ICU, and 71 (37.4%) died in the hospital.

**Table 1:** Patient characteristics according to presence of sarcopenia.

Variables	Pectoralis major		
	Above median CSA (n = 94)	Below median CSA (n = 96)	P-value
Age, years	78 (74, 81)	78 (74, 82)	0.70
Sex, male	56 (59.6%)	57 (60.6%)	1.00
Body mass index (kg/m^2^)	22.6 (20.3, 24.9)	20.7 (17.7, 22.5)	< 0.01
NRS-2002 points, median (IQR)	4 (4, 7)	7 (4, 7)	< 0.01
Intubation	62 (66.0%)	59 (62.1%)	0.57
**Charlson comorbidity index**	3 (2, 4)	2 (1, 4)	0.50
Hypertension	70 (74.5%)	65 (67.7%)	0.34
Diabetes	49 (52.1%)	43 (43.8%)	0.38
Congestive heart failure	10 (10.6%)	9 (9.4%)	0.81
Chronic renal failure	34 (26.2%)	29 (30.2%)	0.44
Chronic obstructive lung disease	11 (11.7%)	11 (11.5%)	1.00
Cancer	16 (17%)	24 (25.0%)	0.21
**Reason for ICU admission**			
Respiratory failure	40 (42.6%)	53 (55.2%)	0.08
Non-respiratory sepsis	27 (28.7%)	25 (26.0%)	0.75
Hemorrhagic shock	4 (4.3%)	2 (2.1%)	0.44
Altered mental status	12 (12.8%)	2 (2.1%)	0.01
Metabolic cause	7 (7.4%)	7 (7.3%)	1.00
Cardiovascular	4 (4.3%)	0 (0.0%)	0.06
Other	0 (0.0%)	7 (7.3%)	0.01
SOFA score at ICU admission, median (IQR)	6 (4, 11)	8 (6, 10)	0.40
Prolonged mechanical ventilation, n (%)*	10/47 (21.3%)	17/40 (42.5%)	0.03
ICU days, median (IQR)	8 (3, 14)	6 (3, 12)	0.59
ICU death, n (%)	15 (16.0%)	28 (29.2%)	0.04
Hospital days, median (IQR)	20.5 (14, 38)	19.5 (12, 41)	0.67
Hospital death, n (%)	26 (27.7%)	45 (46.9%)	0.01

Continuous variables are presented as median (interquartile range) and categorical variables are presented as numbers (percentage).

*CSA* Cross-sectional area, *ICU* intensive care unit, *SOFA* sequential organ failure assessment.

Cutoff values for lower half in pectoralis muscles are 26.5 cm^2^ in men, and 18.3 cm^2^ in women, respectively.

Male patients had higher PM_CSA_ values (P < 0.01). In comparison, patients whose PM_CSA_ values were below the median exhibited a significantly lower body mass index (BMI) (P < 0.01), a higher nutritional risk screening 2002 (NRS-2002) score (P < 0.01), a higher rate of prolonged mechanical ventilation (P = 0.02), and a higher ICU and hospital mortality (P = 0.04 and P = 0.01, respectively) than patients whose PM_CSA_ values were above the median.

### Prolonged mechanical ventilation and SOFA score

In the multivariate logistic regression analysis for the relation of multiple clinical variables with prolonged mechanical ventilation (Table 2), a PM_CSA_ below the median (OR 2.92, 95% confidence interval (95% CI) 1.06–8.06, P = 0.04) and a higher SOFA score at the time of ICU admission (OR 1.20, 95% CI 1.02–1.42, P = 0.03) were related to prolonged ventilation. In the regression analysis for the association of the PM_CSA_ with the longitudinal change of the SOFA score, adjusted for age, sex, BMI, and the Charlson Comorbidity Index (CCI), a PM_CSA_ below the median was related to a higher SOFA score during the ICU stay (estimated mean = 0.94, standard error = 0.44, P = 0.03).

**Table 2:** Clinical factors associated with prolonged mechanical ventilation.

Variables	Odds ratio (95% CI)	P-value
CSA of T4 Pectoralis muscles, below median	2.63 (0.96–7.22)	0.06
Age	0.89 (0.91–1.09)	0.89
Sex, female	1.47 (0.53–4.13)	0.46
Body Mass Index, kg/m^2^	1.02 (0.71–1.02)	0.71
Charlson comorbidity index	0.99 (0.78–1.27)	0.96
SOFA score at the baseline	1.17 (0.78–1.27)	0.06

Data are presented as hazard ratios (95% confidence intervals).

*CSA* Cross sectional area, *SOFA* sequential organ failure assessment.

### Survival and clinical factors associated with mortality

**Figure 1:**
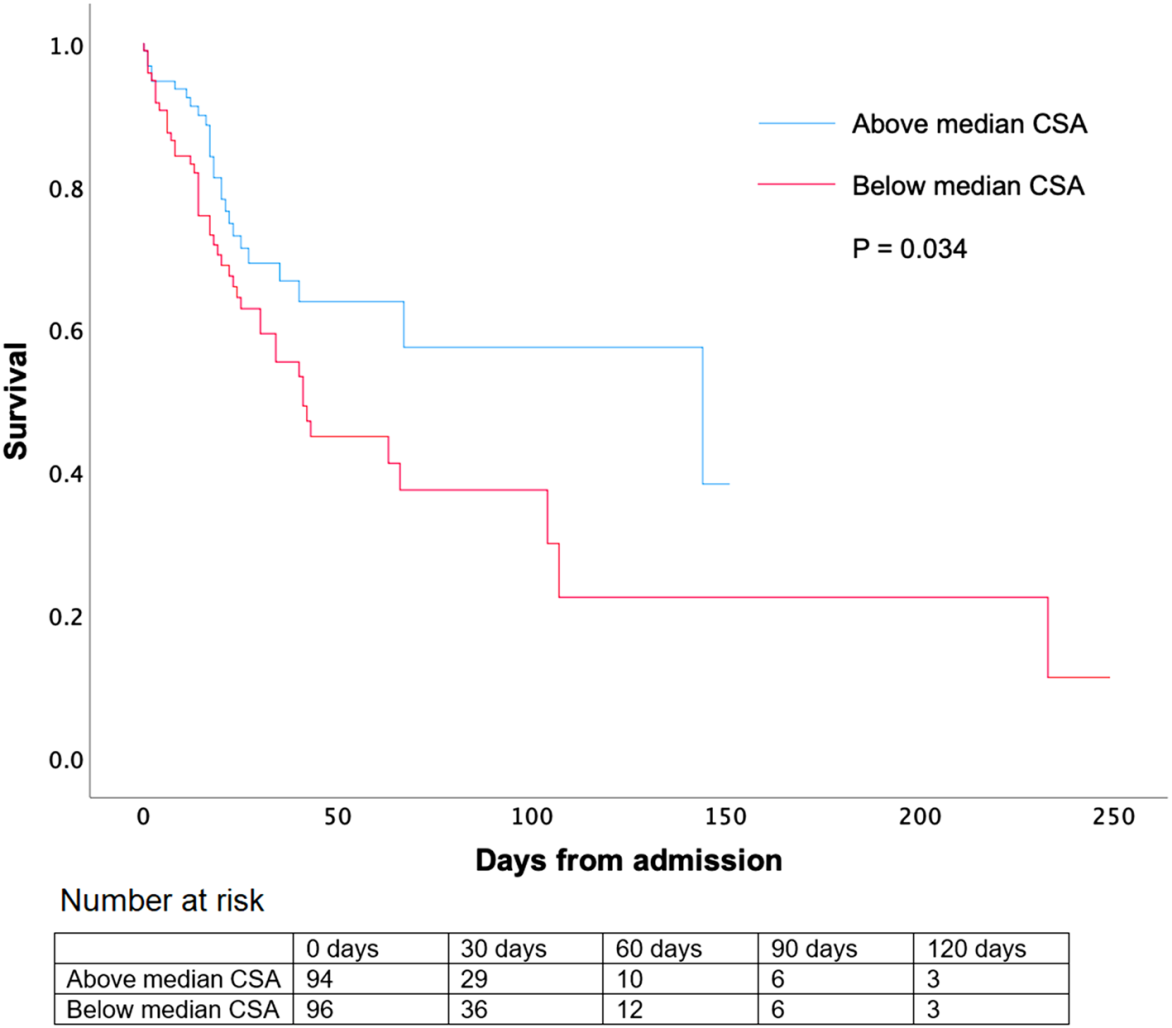
Kaplan–Meier survival curves stratified by the cross-sectional area of muscles at the level of T4 according to the cross-sectional area of the pectoralis muscles. Cutoff values for the lower half in pectoralis muscles are 26.5 cm^2^ in men and 18.3 cm^2^ in women. *CSA* cross-sectional area.

Hospital mortality at 30, 60, and 90 days after ICU admission was 23.4%, 25.5%, and 26.6%, respectively, in patients whose PM_CSA_ values were above the median, whereas it was 34.4%, 41.7%, and 43.8%, respectively, in patients whose PM_CSA_ values were below the median. Kaplan–Meier survival curves stratified by the CSA of the muscles at the level of T4 are shown in Fig. 1; patients were stratified by the PM_CSA_. Patients whose PM_CSA_ values were below the median showed worse survival rates than those whose PM_CSA_ values were above the median (P = 0.034).

The relationships between hospital mortality and clinical parameters were evaluated using Cox proportional hazards analysis (Table 3). Univariate analysis showed that a PM_CSA_ below the median (P = 0.03) and intubation (P < 0.01) were significantly correlated with hospital mortality.

**Table 3:** Clinical factors associated with all-cause mortality.

Variables	Univariate		Multivariate	
	HR (95% CI)	P-value	HR (95% CI)	P-value
CSA of T4 Pectoralis muscles, below median	1.68 (1.04–2.74)	0.03	2.06 (1.23–3.47)	0.01
Age	1.00 (0.96–1.05)	0.99	0.99 (0.95–1.04)	0.79
Sex, Female	0.95 (0.59–1.55)	0.85	0.96 (0.58–1.59)	0.87
BMI, kg/m^2^	0.99 (0.93–1.06)	0.88	1.01 (0.95–1.08)	0.65
Intubation	2.14 (1.23–3.70)	0.01	2.32 (1.33–4.05)	< 0.01
CCI	1.08 (0.98–1.19)	0.12	1.12 (1.02–1.23)	0.02
SOFA score at the baseline	1.07 (0.99–1.15)	0.06	1.1 (1.02–1.18)	0.02

Data are presented as hazard ratios (95% confidence intervals).

*CSA* Cross sectional area, *BMI* body mass index, *CCI* Charlson Comorbidity Index, *SOFA* sequential organ failure assessment.

Additional multivariate Cox proportional hazards analysis was performed to compare the contributions of these indices. In the multivariate analysis, we included the PM_CSA_, age, sex, BMI, CCI, whether the patient underwent intubation, and SOFA score at the time of ICU admission as explanatory variables. Stepwise Cox proportional hazards analysis demonstrated that PM_CSA_ values below the median [hazards ratio (HR) 2.06; 95% CI 1.23–3.47; P = 0.01], intubation (HR 2.32; 95% CI 1.33–4.05; P < 0.01), a higher CCI (HR 1.12; 95% CI 1.02–1.23; P = 0.02), and a higher SOFA score (HR 1.1; 95% CI 1.02–1.18; P = 0.02) were related to hospital mortality.

## Discussion

This study showed that in elderly ICU patients, a low PM_CSA_ at ICU admission was associated with prolonged mechanical ventilation, a higher SOFA score during the ICU stay, and lower mortality.

The present findings agree with previous studies^15,21^, which showed that low CSAs from CTs were correlated with mortality, whereas this study focused on mortality, longitudinal changes in organ dysfunction, namely the SOFA score, and prolonged ventilation. Not only the initial SOFA scores but also the trends in SOFA scores are known to be helpful in predicting the outcomes of ICU patients^22^. Prolonged mechanical ventilation is associated with increased health costs, morbidity, and mortality^23^. As skeletal muscle mass is related to the short- and long-term outcomes in various diseases^24,25^, it would be meaningful to focus on longitudinal changes in prolonged mechanical ventilation and the SOFA scores.

We believe that the CSAs of the pectoralis muscles reflect the patient’s general health and nutritional status and are markers of frailty before ICU admission. Skeletal muscles are important in regulating immune function, glucose disposal, cytokine signaling, and protein synthesis^20^. Skeletal muscle loss is associated with an increased risk of developing nosocomial infections and experiencing falls in the hospital^26^. There are also studies indicating that skeletal muscle loss is associated with depression^27^. It is possible that the factors described above contributed to these findings.

In previous studies on skeletal muscle mass in the ICU using CT as an assessment tool, lumbar muscle at the level of L3 had been the main concern^28,29^. In contrast, this study focused on the thoracic skeletal muscles. According to a previous study, up to 65% of all ICU chest X-rays (CXR) had unexpected or abnormal findings, many of which affected management. The problem is further compounded as CT scanning is being used in critically ill patients who may have multiple medical problems that may not be easily discriminated by the CXR^30^. Without any additional radiation exposure, CSA analysis through existing chest CT scans provides an objective index of future prognosis in elderly ICU patients.

Previous studies^15,19^ included a younger patient population, whereas this study focused on the elderly medical patient population. As the skeletal muscle loss increases with aging and the proportion of elderly patients in the ICU is increasing^1^, it is important to accurately stratify the risk of sarcopenia in elderly ICU patients.

The most appropriate treatment for elderly ICU patients may not necessarily mean maximal treatment, and in patients without improvement in their clinical situation, the therapeutic intensity level may no longer be in accordance with patients’ chances of long-term survival with acceptable quality of life, during which a clinical decision might need to be made^2^. In the algorithm on the decision-making process for caring for critically ill older patients proposed by Bertrand et al.^2^, it is recommended to assess the patient’s condition and arrange a meeting with the family on day 2 or 3 of the ICU admission. Considering the thoracic skeletal muscle CSA at the time of ICU admission when deciding the therapeutic intensity for elderly ICU patients may help in making medical decisions.

In abdominal CT, the muscle area at the L3 vertebra level, divided by the patient height^2^, is accepted as a surrogate marker of loss of skeletal muscle quantity and cachexia^31^. However, at the thoracic level, the method of assessing skeletal muscle loss has not been established, and the muscle areas measured for each study were different. For example, a study by Ariel et al.^15^ assessed the pectoralis muscle CSA, a study by Florian et al.^32^ assessed the thoracic skeletal muscles at the level of T5, a study by Lee et al.^33^ assessed diaphragm thickness, and a study by Fuseya et al.^10^ used the erector spinae muscle CSA at the level of T12. In this study, we analyzed the CSA of the pectoralis at the level of T4. Muscles at the level of T4 have shown clinical significance in various diseases^12,14,34^; among these muscles, the pectoralis muscle CSA at the level of T4 is easy to identify, and its area can be standardized across cohorts^12^. However, more studies are needed to clarify which muscles among the thoracic muscles best reflect sarcopenia.

Our study has limitations. First, the nature of this study was retrospective. Second, the study population was from a single center, consisted only of Asian patients, and involved no replication cohort. Thus, our results may not be fully generalizable to other populations. However, validity of the reported prognostic factors in ICU patients, such as the CCI and SOFA scores, was confirmed in this study population, which supported the present findings. Third, the physical activity levels of all subjects were not directly evaluated. We could not include data regarding physical function testing. However, based on other reports^6,7,11^, we suspected that the skeletal muscles of the chest may reflect both the physical activity and physiological parameters. Further analysis is needed to verify these assumptions. Fourth, we could not perform pectoralis muscle mean density measurements because less than half of the patients received non-contrast CTs.

In conclusion, low CSAs of the pectoralis muscle obtained from single-slice axial chest CT were associated with prolonged mechanical ventilation, higher SOFA scores during ICU stays, and a higher mortality. Considering the thoracic skeletal muscle CSA at the time of ICU admission when deciding the therapeutic intensity in elderly ICU patients may help in making medical decisions.

## Methods

### Study design and population

We retrospectively reviewed the medical records of patients aged > 70 years admitted to the ICU between August 2016 and December 2018 in a tertiary care hospital in South Korea. The flow diagram of the subjects in this study is depicted in Supplementary Fig. 3. Initially, 1085 patients who were admitted to the medical ICU were included. Patients were excluded if (1) the patient was admitted from another hospital or ICU; (2) the patient did not perform a chest CT scan within two days before ICU admission; (3) the patient was aged < 70 years; (4) data on the performance status and death were unavailable; or (5) the quality of the chest CT scan was poor. After meeting these criteria, 190 (113 men and 77 women) subjects were included in the analysis. As our hospital operates a cardiac ICU, an oncology ICU, a pediatric ICU, and a surgical ICU, patients from these units were not included in our study.

Age, body mass index (BMI), underlying diseases (CCI), NRS-2002 points, mechanical ventilation, ICU and hospital discharge, the serial weekly SOFA score up to 4 weeks, and mortality data were collected for all patients; data regarding the pectoralis muscle cross-sectional area at the level of the fourth thoracic vertebra (PM_CSA_) were available in all patients. The NRS-2002 used in this study was calculated according to the study by Kondrup et al.^35^. The CCI used in this study was calculated according to the study by Charlson et al.^36^. The SOFA score was calculated according to the study by Jones et al.^37^. Prolonged mechanical ventilation was defined as greater than 21 days of mechanical ventilation required for at least six hours per day^38^. Follow-up data, including mortality data, were collected until June 2019. This study was performed in accordance with the provisions of the Declaration of Helsinki. This research protocol was approved by the Institutional Review Board of Severance Hospital. (IRB No. 2019-2889-001) The study design was approved by the appropriate ethics review boards. Due to the retrospective nature of this study, the requirement to obtain informed patient consent was waived. (The Institutional Review Board of Severance Hospital waived the need to provide informed consent).

### Measurement of CSA of the skeletal muscle at the level of T4

**Figure 2:**
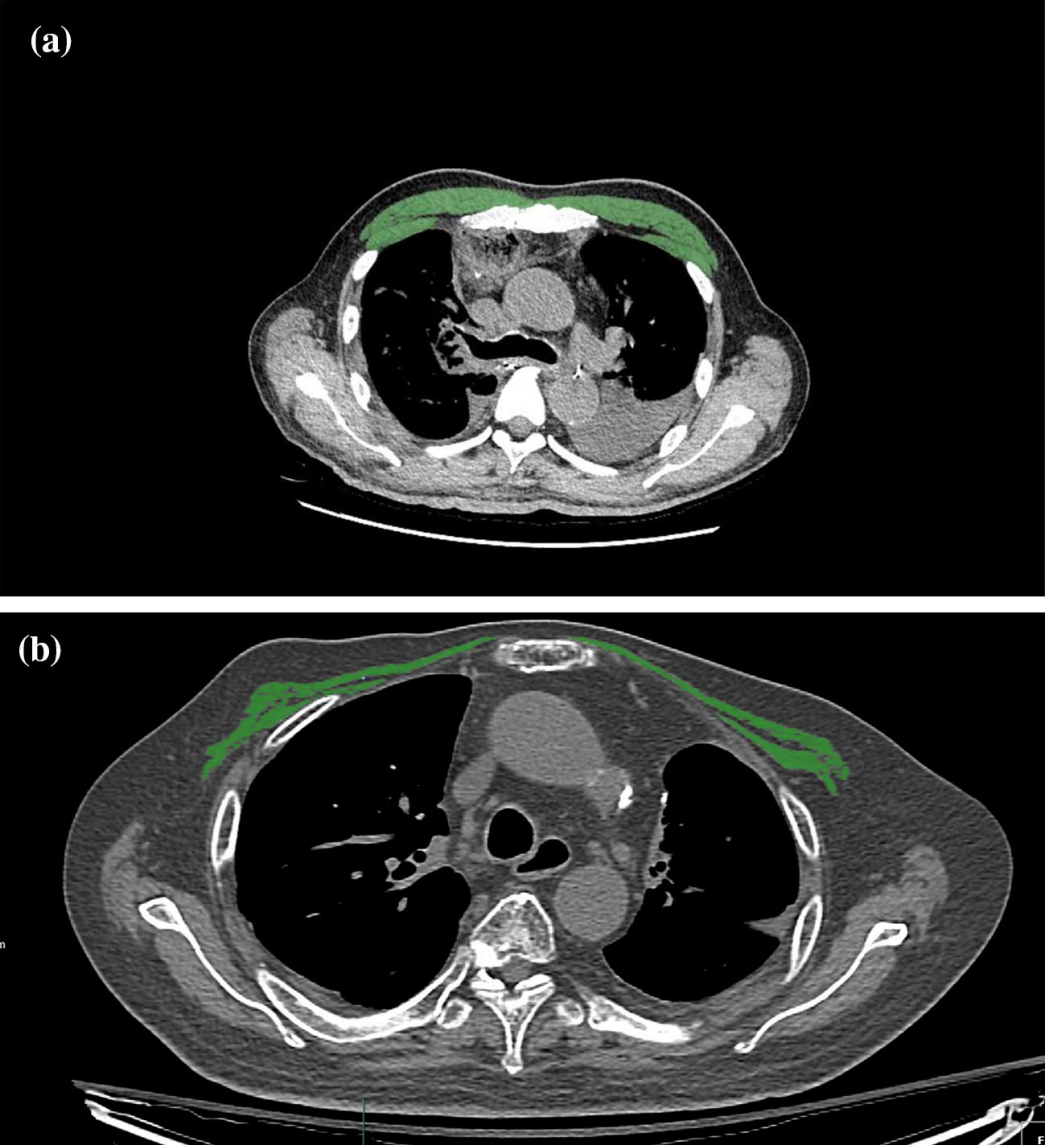
Sample axial computed tomography (CT) images of the fourth thoracic vertebral region. (**a**) A patient with PM_CSA_ values above the median. (**b**) A patient with PM_CSA_ values below the median. Images are used to determine the muscle area in elderly ICU patients. The pectoralis muscles are shown in green.

CT within 2 days before the admission to the ICU was used for the analysis because approximately 10% of the total body muscle mass may be lost in hospitalized elderly patients from only 3 days of immobility^39^. All scans were performed with the following scanning parameters: a tube voltage of 120 kVp, an average tube current of 300 mA, an average pitch of 0.9, and a volume CT dose index less than 6.0 mGy. After scanning, axial images were reconstructed at a slice thickness of 1 mm and a slice increment of 1 mm with a medium-smooth convolution kernel. Measurements of the major and minor pectoralis muscles were performed as in our previous study^15^. Quantitative assessment of the CSA was performed semi-automatically using the Aquarius iNtuition Viewer (ver. 4.4.11, TeraRecon Inc., San Mateo, CA, USA), as shown in Fig. 2. The T4 level was defined as the slice including the middle of the fourth thoracic vertebrae by comparing the sagittal and transverse views; the observers visually identified single cross-sectional images in which the borders of the thoracic and back muscles were outlined. Tissue CSAs in slices were computed automatically by summation of the pixel attenuation of − 30 to + 150 Hounsfield units for skeletal muscle. After applying the threshold method (with a predefined Hounsfield unit threshold) to slices, boundaries between different tissues were manually corrected. Contrast-enhanced and non-contrast CT scans were used, as there was no difference in muscle CSA measurements between these in the previous study^40^. The measurement of the CSAs was performed independently by two radiology technicians with 4 and 6 years of experience. Differences greater than 5% were settled by a third radiology technician who had 10 years of experience. Radiology technicians performed measurements of the CSAs without access to patient information. CSA values below the median value in the respective sexes were considered to indicate loss of skeletal muscle mass.

### Statistical analysis

Descriptive statistics are reported as numbers with proportions or medians with interquartile ranges. Fisher's exact tests were conducted to compare categorical variables between the survivor and non-survivor groups; Mann–Whitney tests were conducted to compare continuous variables between the two groups. To analyze the effect of muscle CSAs on the SOFA score, the linear mixed model analysis for the continuous longitudinal dataset was used. To evaluate the relationship between prolonged mechanical ventilation and multiple clinical parameters while controlling potential confounding factors, multivariate logistic regression models were used. Survivals were estimated with the Kaplan–Meier method and compared with the log-rank test. Multivariate Cox proportional hazards models were performed to investigate relationships between clinical parameters and mortality. NRS-2002 points were excluded from the multivariate analyses since the BMIs were included in the calculation of the NRS-2002 points. An adjusted *P*-value < 0.05 was considered statistically significant. All statistical analyses were performed with SPSS version 25.0 (SPSS Inc., Chicago, IL, USA, https://www.ibm.com/support/pages/downloading-ibm-spss-statistics-25).

## Supplementary Information

Supplementary Figures.

## Data Availability

The datasets used and/or analyzed are available from corresponding author upon reasonable request.
