# Supplementary material for: Thoracic skeletal muscle quantification using computed tomography and prognosis of elderly ICU patients
Source: Sci Rep. 2021 Dec 6;11:23461. doi: 10.1038/s41598-021-02853-4 (PMC8648749; doi:10.1038/s41598-021-02853-4)
Supplement: Supplementary file 1 — Supplementary Figures. [file 41598_2021_2853_MOESM1_ESM.docx]

**SUPPLEMENTARY INFORMATION**

**Thoracic skeletal muscle quantification using computed tomography and prognosis of elderly ICU patients**

**Authors:**

Sung Woo Moon, Song Yee Kim, Ji Soo Choi, Ah Young Leem, Su Hwan Lee, Moo Suk Park, Young Sam Kim, Kyung Soo Chung

**Figure legends**

**Supplementary Fig 1. Distribution of cross-sectional area of pectoralis muscles.**


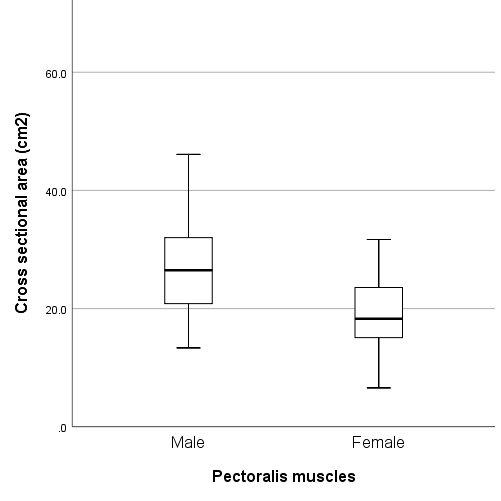


Median values for CSA of pectoralis muscles are 26.5 cm^2^ in men, and 18.3 cm^2^ in women, respectively.

**Supplementary Fig 2. SOFA score changes during the follow up period.**


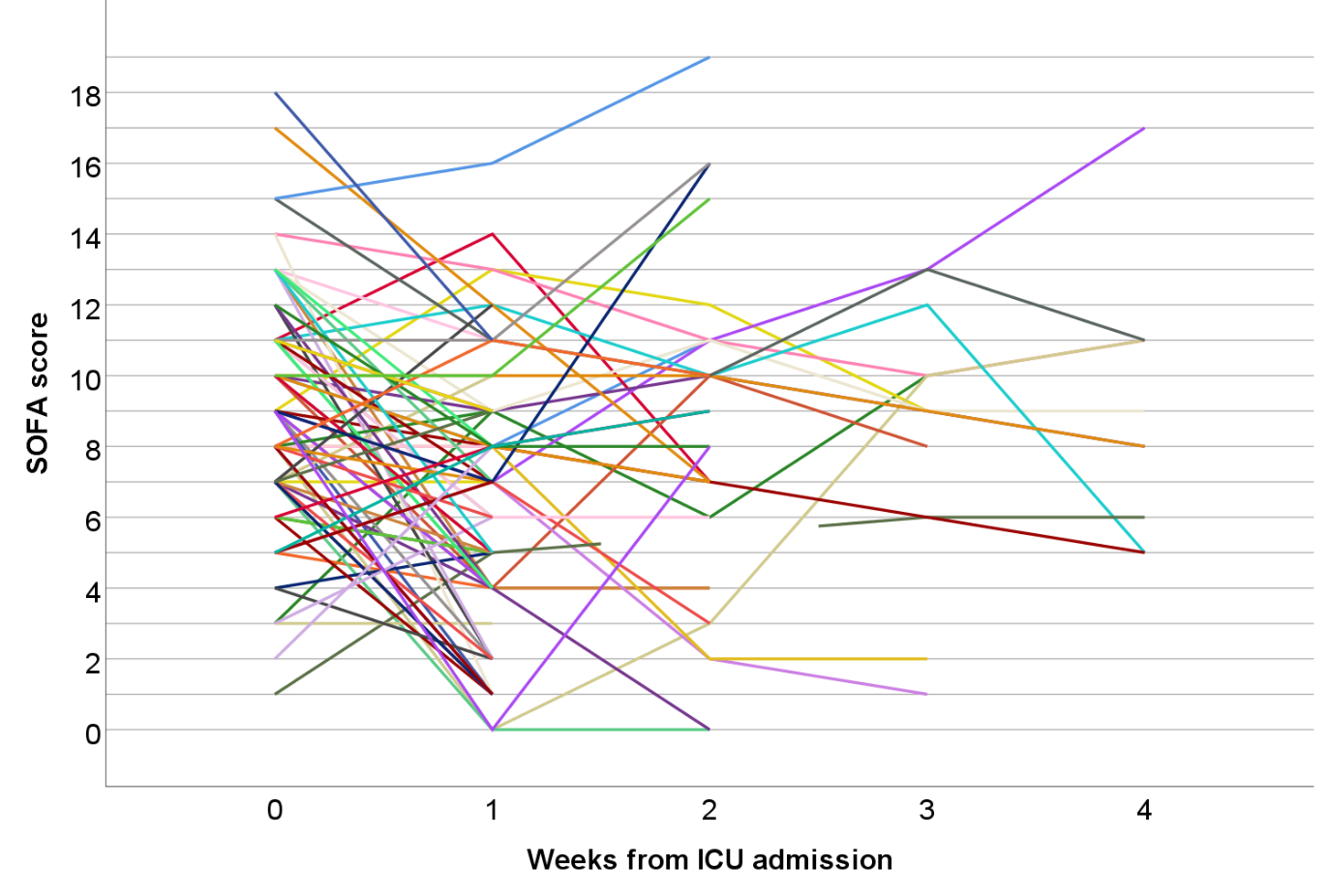


Abbreviations; SOFA, sequential organ failure assessment

**Supplementary Fig 3. Flow diagram of subjects in this study.**

Abbreviations; ICU, intensive care unit; CT, computed tomography; PM_CSA_, cross-sectional area of pectoralis muscle area at the T4 level; T4_CSA_, cross-sectional area of pectoralis, intercostalis, paraspinals, serratus, and latissimus muscles at the T4 level.

**
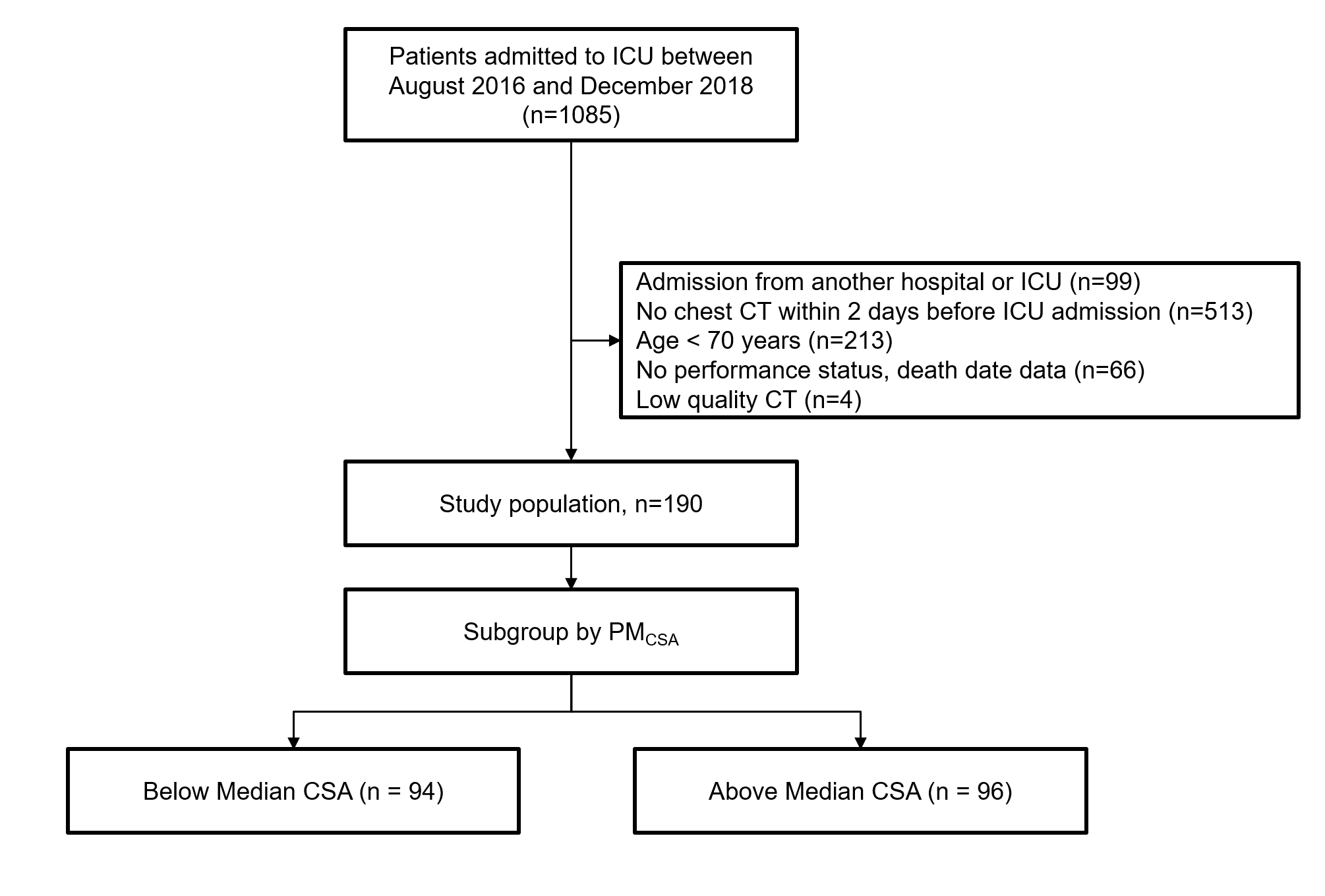
**
